# The role of m^6^A and m^6^Am RNA modifications in the pathogenesis of diabetes mellitus

**DOI:** 10.3389/fendo.2023.1223583

**Published:** 2023-07-07

**Authors:** Daniel Benak, Stepanka Benakova, Lydie Plecita-Hlavata, Marketa Hlavackova

**Affiliations:** ^1^ Laboratory of Developmental Cardiology, Institute of Physiology of the Czech Academy of Sciences, Prague, Czechia; ^2^ Department of Physiology, Faculty of Science, Charles University, Prague, Czechia; ^3^ Laboratory of Pancreatic Islet Research, Institute of Physiology of the Czech Academy of Sciences, Prague, Czechia; ^4^ First Faculty of Medicine, Charles University, Prague, Czechia

**Keywords:** type 2 diabetes mellitus, T2DM, diabetes, RNA, epigenetics, epitranscriptomics, m6A, m6Am

## Abstract

The rapidly developing research field of epitranscriptomics has recently emerged into the spotlight of researchers due to its vast regulatory effects on gene expression and thereby cellular physiology and pathophysiology. N^6^-methyladenosine (m^6^A) and N^6^,2’-O-dimethyladenosine (m^6^Am) are among the most prevalent and well-characterized modified nucleosides in eukaryotic RNA. Both of these modifications are dynamically regulated by a complex set of epitranscriptomic regulators called writers, readers, and erasers. Altered levels of m^6^A and also several regulatory proteins were already associated with diabetic tissues. This review summarizes the current knowledge and gaps about m^6^A and m^6^Am modifications and their respective regulators in the pathophysiology of diabetes mellitus. It focuses mainly on the more prevalent type 2 diabetes mellitus (T2DM) and its treatment by metformin, the first-line antidiabetic agent. A better understanding of epitranscriptomic modifications in this highly prevalent disease deserves further investigation and might reveal clinically relevant discoveries in the future.

## 1 Introduction

Diabetes mellitus is one of the most common chronic diseases with an increasing prevalence (1). Type 2 diabetes mellitus (T2DM) is more frequent than type 1 diabetes mellitus (T1DM) and accounts for approximately 90% of all cases of diabetes (2). This heterogeneous systemic disorder is mainly characterized by two factors: deficient insulin secretion by pancreatic β-cells and insulin resistance of insulin-sensitive tissues (3). The subsequent chronic hyperglycemia, a hallmark of T2DM, damages glucose-sensitive organs and results in downstream deficits in vital functions (4). Despite a considerable amount of data collected regarding T2DM, the molecular mechanism of its development is still unclear. However, it is known that T2DM is linked with the dysregulation of gene expression profiles in cells (5–7). Epitranscriptomic modifications of RNA are one of the possible mechanisms by which gene expression could be affected during the pathogenesis of T2DM.

**Figure 1:**
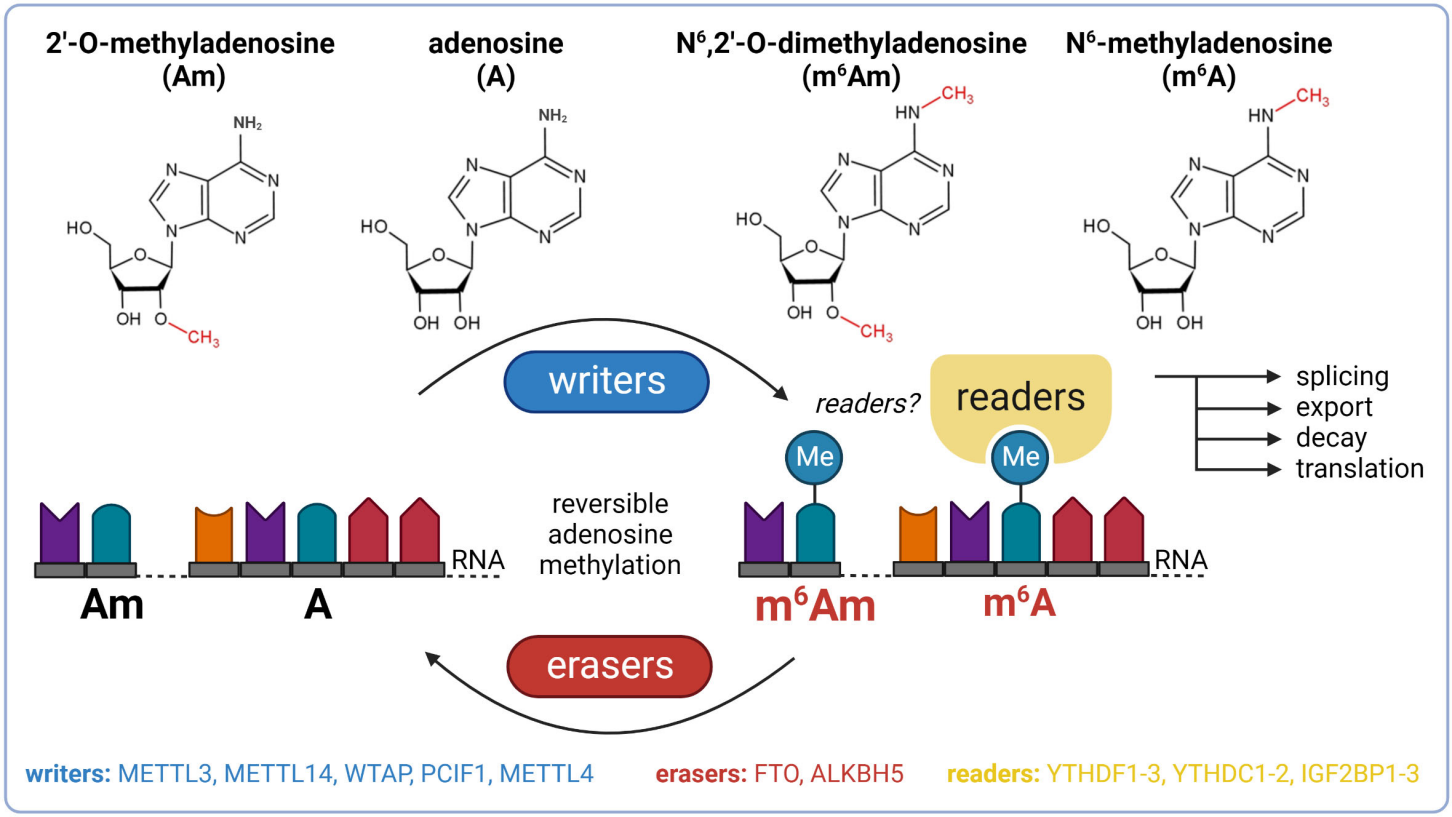
Basic overview of m^6^A and m^6^Am epitranscriptomics. ALKBH5, AlkB family member 5; FTO, fat mass and obesity-associated; IGF2BP1-3, insulin-like growth factor 2 mRNA binding proteins 1-3; METTL3, methyltransferase-like 3; METTL14, methyltransferase-like 14; WTAP, Willms’ tumor 1-associating protein; YTHDC1-2, YTH domain-containing protein 1-2; YTHDF1-3, YTH domain-containing family proteins 1-3.

To date, over 170 chemical modifications have been described in RNA (8). N^6^-methyladenosine (m^6^A) and N^6^,2’-O-dimethyladenosine (m^6^Am) are among the most prevalent and well-characterized RNA-modified nucleosides (9–12). The biological effects of these modifications are regulated by proteins called writers (methylation deposition), readers (binding of modified RNA), and erasers (methylation removal). The presence or absence of m^6^A and m^6^Am in mRNA affects key stages of its life cycle, including splicing, export, decay, and translation (**Figure 1**) (13, 14). These dynamic modifications with profound impact on gene expression regulation might thereby play an important role in the pathogenesis of T2DM and become the future targets in the search for the next generation of anti-diabetic drugs.

## 2 N^6^-methyladenosine

The most prevalent modification in eukaryotic mRNA is m^6^A (9, 10). Besides mRNA, m^6^A also occurs in other types of RNA, including ribosomal RNA (rRNA), long non-coding RNA (lncRNA), small nuclear RNA (snRNA), or microRNA (miRNA) (15). The deposition of the methyl group to adenosine (A) is performed by a multicomponent methyltransferase complex (MTC) with a stable core component formed between methyltransferase-like 3 (METTL3) and methyltransferase-like 14 (METTL14). METTL3 functions as a catalytic subunit and METTL14 facilitates RNA binding (16, 17). The third major component of the MTC is the Willms’ tumor 1-associating protein (WTAP) which interacts with the METTL3/METTL14 heterodimer and promotes the localization of the MTC to nuclear speckles (18). The reverse process, demethylation of m^6^A back to A, is mediated by enzymes called demethylases. In 2011, Fat mass and obesity-associated protein (FTO) was the first described demethylase of m^6^A (19). This discovery provided evidence of reversible posttranscriptional modifications in mRNAs and renewed the interest of researchers in mRNA modifications (20). After 2 years, alkB homolog 5 (ALKBH5) was reported as another m^6^A eraser (21). The biological functions of m^6^A can be mediated by m^6^A readers which recognize and selectively bind to m^6^A-decorated RNAs. The most prominent readers are YTH domain-containing family proteins 1-3 (YTHDF1-3) which mediate the degradation of methylated mRNAs, and YTH domain-containing proteins 1-2 (YTHDC1-2) which regulate mRNA splicing and facilitate translation initiation (22–28). In addition to YTH proteins, other readers described include insulin-like growth factor 2 mRNA-binding proteins 1-3 (IGF2BP1-3) which promote the stability of their target mRNAs in an m^6^A-dependent manner under normal and stress conditions and therefore also affect gene expression output (29).

## 3 N^6^,2’-O-dimethyladenosine

m^6^Am is another prevalent form of modified adenosine, but it is much less studied than m^6^A. This modification is formed by the methylation of a 2’-O-methyladenosine (Am). It has been described only in mRNA and snRNA. In mRNA, m^6^Am is found directly downstream to the 7-methylguanosine (m^7^G), forming the extended cap structure (11, 12). It has been found in at least 30-40% of all transcripts in vertebrate mRNA (11). However, in specific cell lines, m^6^Am is even more dominant. For instance, HEK293T cells have 92% of 5’ capped mRNAs with m^6^Am and only 8% with single methylated Am (30). The presence of m^6^Am in mRNA markedly enhances its stability (31). In snRNA, m^6^Am is also present at its internal sites and influences pre-mRNA splicing (11, 32). N^6^-methylation of Am to m^6^Am is catalyzed by two known writers: phosphorylated CTD interacting factor 1 (PCIF1) and methyltransferase-like 4 (METTL4). PCIF1 has been described as a cap-specific adenosine-N^6^-methyltransferase (also called CAPAM) which does not methylate adenosine residues in the RNA body (30, 33). However, recently it was reported that PCIF1 also has ancillary methylation activities on internal adenosines (both A and Am), although with lower affinities (34). Importantly, before the recognition of methyltransferase activity of PCIF1, this protein was known to inhibit pancreatic and duodenal homeobox protein 1 (PDX1), a transcription factor crucial for normal pancreas development and function (35, 36). METTL4, the second methyltransferase, is responsible for internal m^6^Am formation within U2 snRNA (37, 38). The only described m^6^Am eraser so far is FTO, the well-known m^6^A demethylase. In 2017, it was reported that FTO preferentially demethylates m^6^Am rather than m^6^A (31, 39), but recent studies suggested that the substrate preference of FTO might depend on its cellular localization which varies between cell types. In the nucleus, FTO preferably targets m^6^A whereas cytosolic FTO demethylates especially m^6^Am (40, 41). Thus, special attention is needed in FTO research to distinguish the m^6^A- and m^6^Am-specific effects of this demethylase (42). No readers of m^6^Am have been described so far.

## 4 Pathogenesis of T2DM: the role of m^6^A and m^6^Am modifications

### 4.1 Genetic predisposition to T2DM

The development of T2DM is the result of interaction between environmental factors (e.g. unhealthy diet, sedentary lifestyle, stress) and a strong hereditary component (43). Currently, several hundreds of genetic variants were associated with T2DM, although mostly with only minor effects on disease development (44).

Numerous studies suggested that m^6^A and m^6^Am demethylase *FTO* is among the genes whose variants possess the highest genetic risk of T2DM (44). However, this link is still controversial with significant interethnic differences (45, 46). For instance, the common *FTO* rs9939609 variant was associated with T2DM in white American, Palestinian, Asian Indian, and obese Iraqi populations, but not in Bengalee Hindu, North Indian, nor Saudi populations (47–57). Also, other genetic polymorphisms in the *FTO* gene were identified as T2DM risk factors. Carriers of the *FTO* rs17817449 variant in the Czech-Slavonic and obese Iraqi populations were more susceptible to T2DM and chronic diabetic complications (44, 51, 58). In Iranian obese women, *FTO* variants rs763967273, rs759031579, rs141115189, rs9926289, rs76804286, and rs9939609 were all related to T2DM (59). On the contrary, African-Americans carrying the rs1421085 C allele were found to be protected against diabetes (54). The polymorphisms in *FTO* gene seem to regulate the expression level of FTO and its enzymatic function. Detrimental effects of high or low expression of FTO were already confirmed in experimental studies. For instance, it has been shown that FTO depletion activates inflammatory response, one of the main pathogenic features in T2DM patients (60).

Besides *FTO*, variants of *IGF2BP2*, an m^6^A reader, were also associated with a significant risk of T2DM development, namely variant rs4402960 in Asian Indian Sikhs, Czechs, or Italians, and rs11705701 in the Chinese population (44, 55, 61, 62).

Although further studies are needed to unravel the complex polygenic background of T2DM, it seems to be clear that genetic polymorphisms in genes encoding epitranscriptomic regulators are associated both with T2DM and its complications.

### 4.2 Pancreatic islets

**Figure 2:**
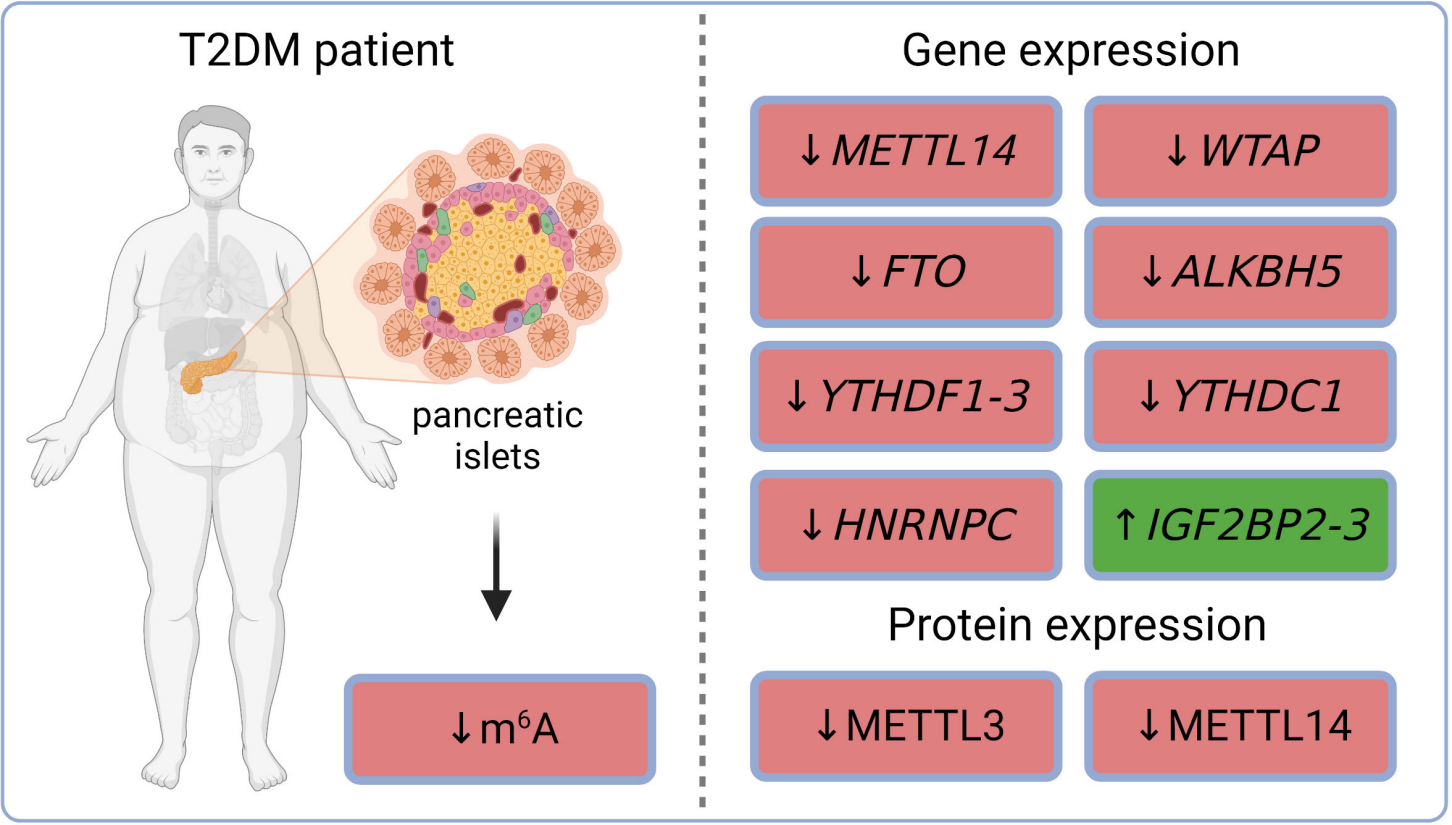
m^6^A and m^6^Am regulations in pancreatic islets of T2DM patients. ALKBH5, AlkB family member 5; FTO, fat mass and obesity-associated; HNRNPC, heterogeneous nuclear ribonucleoprotein C; IGF2BP2-3, insulin-like growth factor 2 mRNA binding proteins 2-3; m^6^A, N^6^-methyladenosine; METTL3, methyltransferase-like 3; METTL14, methyltransferase-like 14; T2DM, type 2 diabetes mellitus; WTAP, Willms’ tumor 1-associating protein; YTHDC1, YTH domain-containing protein 1; YTHDF1-3, YTH domain-containing family proteins 1-3.

Pancreatic β-cell failure mediated by metabolic stress is the central event in the pathogenesis of T2DM (63). Although the mechanisms underlying β-cell dysfunction are still not fully understood, emerging data suggest an involvement of epigenetic modifications in the adaptation of β-cells to metabolic stress (64).

m^6^A sequencing in dispersed islets from controls and T2DM patients revealed 6,078 differently methylated sites in 4,155 mRNAs and a higher number of sites with decreased levels of m^6^A methylation in T2DM compared to controls. Gene ontology analysis of the m^6^A methylome revealed that the genes affected in T2DM patients are involved in cell-cycle regulation, receptor signaling, insulin secretion, and pancreas development (65). The decreased total m^6^A levels were observed in Langerhans islets of T2DM patients and also in islets of mice fed with a high-fat diet (a model mimicking T2DM phenotype). Similarly, high glucose conditions (state typical for T2DM) also resulted in lower methylation levels in non-diabetic human pancreatic islets as well as in mouse β-cell line (Min6) (66). Gene expression analysis in whole islets collected from healthy humans and patients with T2DM revealed a down-regulation of several m^6^A regulators in diabetic individuals – methyltransferase *METTL14*, demethylases *FTO* and *ALKBH5*, and readers *YTHDF1* and *YTHDF3*. In addition to transcripts, protein levels of methyltransferases METTL3 and METTL14 were also decreased (65). The reduction of *FTO* gene expression and METTL3/14 protein levels in T2DM human islets was observed also in other studies (67–69). RNA-seq datasets (GSE153855; GSE153855) from T2DM and non-T2DM individuals revealed increased gene expression of readers *IGF2BP2-3* and decreased gene expression of writer *WTAP* and readers *YTHDF2-3*, *YTHDC1*, and *HNRNPC* (70–72). m^6^A reader *IGF2BP2* was also up-regulated in β-cells obtained from cadaver pancreases of T2DM patients (73). The current knowledge of diabetic epitranscriptomic changes in human Langerhans islets is summarized in **Figure 2**. Overall, it seems that the whole epitranscriptomic machinery is attenuated in human diabetic islets. The only up-regulated genes *IGF2BP2-3* have also functions unrelated to epitranscriptomics, which might explain their opposite trend.

In contrast to these results, Bornaque et al. (66) showed that high glucose concentrations in Min6 cells increased mRNA expression of important m^6^A regulators – methyltransferase *Mettl3* and demethylases *Fto* and *Alkbh5*. Glucose treatment also induced a shift in the subcellular protein localization of METTL3 and ALKBH5 (66). Overexpression of *FTO* in Min6 cells promoted the production of reactive oxygen species (ROS) and led to NF-κB activation, which resulted in the inhibition of insulin secretion (74). These differences between a specific mouse cell line and heterogeneous human islets might be explained by interspecies variation or islet heterogeneity.

MTC specifically regulates the postnatal functional maturation of β-cells. Mice with deletion of *Mettl3/14* in Ngn3^+^ endocrine progenitor cells developed hyperglycemia and hypoinsulinemia 2 weeks after birth. This study also showed that *Mettl3/14* deletion silenced the expression of important transcription factors, such as *Mafa*, *Nkx6-1*, or *Pdx1* (69). Other studies using mouse models with β-cell-specific deletions of MTC subunits (*Mettl3*, *Mettl14*, *Wtap*) also pointed out the importance of MTC in maintaining β-cell function. Deletion of either subunit resulted in decreased m^6^A levels (65, 70, 75). METTL3 deficiency led to β-cell failure and hyperglycemia (75). METTL14-deficient mice exhibited decreased β-cell mass, reduced insulin secretion, and glucose intolerance (65, 76, 77). Deficiency of WTAP was associated with a reduction of METTL3 levels and resulted in severe hyperglycemia and β-cell failure. Overexpression of *Mettl3* in β-cells partially prevented the negative effects of WTAP deficiency (70). Comparing *Mettl3*-βKO and *Wtap*-βKO mice revealed down-regulation of β-cell-specific transcription factors (such as *Mafa*, *Nkx6-1*, *Pdx1*, *Neurod1*, or *Foxa2*) and insulin secretion-related genes (such as *Ins1*, *Ins2*, *Brsk2*, *Cacna1c*, *Doc2b*, *Ffar1*, *G6pc2*, *Gck*, *Gipr*, *Hadh*, *Ica1*, *Nnat*, *Park7*, *Pclo*, *Selenot*, *Serp1*, *Slc30a8*, *Stxbp51*, *Sytl4*, *Trpm2*, *Ucn3*, and *Uqcc2*) (70). Besides methyltransferases, also β-cell-specific deletion of reader *Ythdc1* resulted in β-cell failure and diabetes (71, 78). This was likely due to the decreased gene expression of β-cell-specific transcription factors (such as *Mafa*, *Nkx6-1*, *Neurod1*, and *Hmgn3*) and insulin-related genes (such as *Ins1*, *Ins2*, *Gck*, *G6pc2*, *Sytl4*, *Doc2b*, *Pclo*, *Cacna1c*, *Slc30a8*, *Ffar1*, *Gipr*, *Nnat*, and *Selenot*). Transcription factor MAFA decreased dramatically also on protein level in *Ythdc1*-βKO islets (71). Yang et al. suggested that YTHDC1 may regulate mRNA splicing and export to modulate glucose metabolism in β-cells by interacting with serine/arginine-rich splicing factor 3 (SRSF3) and cleavage and polyadenylation specific factor 6 (CPSF6) (78).

These data indicate that m^6^A/m^6^Am epitranscriptomic machinery vastly affects the biology of pancreatic β-cells and plays a role in the induction of diabetic phenotype. However, the data are still fragmental, and more studies covering more m^6^A/m^6^Am regulators are needed to elucidate the exact role of epitranscriptomic regulations in the diabetic pancreas.

### 4.3 Heart

Cardiovascular disease (CVD) is a common comorbidity and a major cause of mortality among people with T2DM. More than 30% of all T2DM patients are affected by CVD (79). Cardiac dysfunction observed in patients with diabetes that occurs in the absence of other cardiovascular risk factors (such as hypertension, coronary artery disease, or valvular disease) is referred to as diabetic cardiomyopathy (DCM) (80). This condition is characterized by cardiac diastolic dysfunction and later by heart failure (HF) and cardiac death. It is estimated that the risk of HF is 2-3 times higher in individuals with T2DM and that approximately 12% of diabetic patients eventually develop severe HF often leading to death (81). The epitranscriptomic modifications, including m^6^A, are known to play various roles in the physiology and pathophysiology of the cardiovascular system (20, 82–85). Recent studies have shown that changes in m^6^A methylation also contribute to HF progression (86–90). However, the role of cardiac m^6^A and m^6^Am machinery is not well-characterized in T2DM.

**Figure 3:**
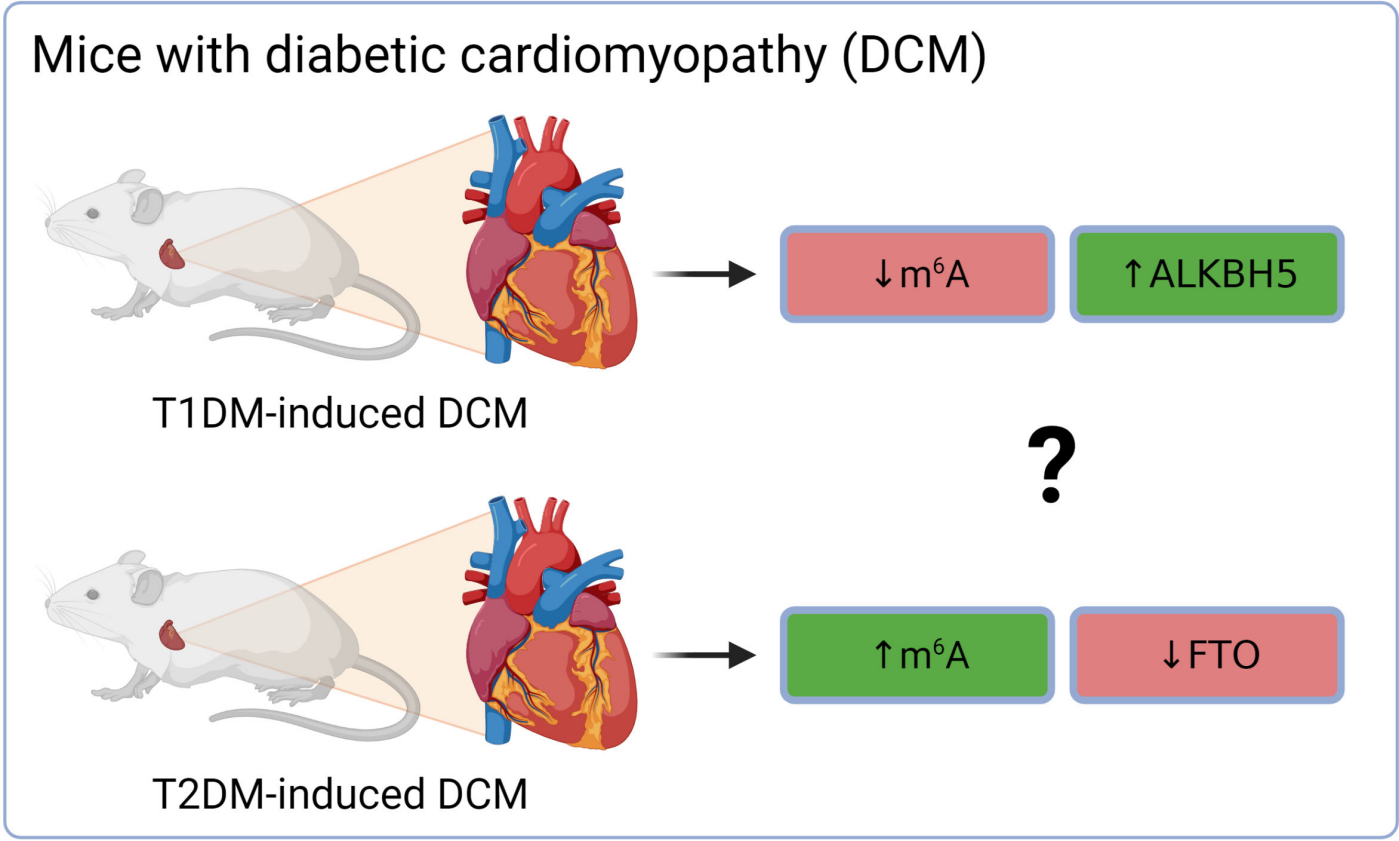
Different epitranscriptomic regulations in DCM on T1DM and T2DM mouse hearts. ALKBH5, AlkB family member 5; FTO, fat mass and obesity-associated; m^6^A, N^6^-methyladenosine; T1DM, type 1 diabetes mellitus; T2DM, type 2 diabetes mellitus.

Altered cardiac m^6^A patterns were detected in db/db mice (model of T2DM and DCM). The differentially methylated transcripts were linked mainly to cardiac fibrosis, myocardial hypertrophy, and myocardial energy metabolism (91). The higher total m^6^A mass in DCM was associated with the down-regulation of demethylase FTO on both gene and protein levels, while levels of METTL3, METTL14, and ALKBH5 were stable (91). Interestingly, mice with T1DM-induced DCM (C57BL/6 mice injected with streptozotocin) exhibited a different dysregulation of epitranscriptomic machinery (**Figure 3**). Total m^6^A levels in the hearts of these mice were decreased. This was linked with an increase of ALKBH5 in the cardiomyocytes of DCM mice and subsequent activation of the Hippo signaling pathway through a YTHDF2-dependent action (92). These results suggest that the two types of diabetes might affect the epitranscriptomic background of DCM differently. It has been reported already that T1DM and T2DM might affect the heart in a different way and result in dissimilar DCM phenotype. This was explained mainly by the different myocardial insulin action (insulin deficiency in T1DM vs insulin resistance and hyperinsulinemia in T2DM) and thus distinct signaling downstream of the insulin receptor (93). Therefore, the contradictory epitranscriptomic results may be explained by the different phenotype between the two types of diabetes. However, further research is needed to resolve this issue.

Most of the studies dealing with m^6^A/m^6^Am regulations in DCM have been executed on T1DM animal models. Pyroptosis, a type of proinflammatory cell death, is tightly involved in DCM progression. Methyltransferase METTL14 was down-regulated in the hearts of rats with DCM (T1DM-induced) and enhancement of its expression inhibited pyroptosis in myocardial tissues and improved systolic function (increased fractional shortening and ejection fraction) via down-regulation of lncRNA *Tincr*. The expression of *Tincr* was regulated in a YTHDF2-dependent manner (94). Peng et al. (95) reported that lncRNA *Airn* ameliorated diabetes-induced (T1DM) cardiac dysfunction caused by cardiac fibrosis. Their data showed that *Airn* binds to m^6^A reader IGF2BP2 and protects it from ubiquitin-proteasome-dependent degradation, leading to an m^6^A-dependent stabilization of *p53* mRNA by IGF2BP2 and subsequent reduction in cardiac fibrosis (95).

Despite the limited amount of data available, it is becoming evident that epitranscriptomic dysregulations in diabetic cardiac tissue might have a significant effect on the function of the heart. However, the exact role of m^6^A and m^6^Am in DCM induced by each type of T2DM is yet to be deciphered.

### 4.4 Kidneys

Diabetic nephropathy (DN), also known as diabetic kidney disease, is a prevalent microvascular complication of T2DM often leading to end-stage renal disease, a life-threatening condition (96). According to the International Diabetes Federation reports, up to 40% of diabetic patients might develop DN (97).

Xu et al. reported, that human kidney 2 (HK-2) cells stimulated with high glucose decreased total m^6^A methylation level and also methyltransferases METTL3 and METTL14 (98). Interestingly, Jiang et al. observed increased m^6^A modification in diabetic mice which was caused by elevated levels of METTL3. They also found increased METTL3 levels in renal biopsies from DN patients. Further experiments showed that METTL3 exerted pro-inflammatory and pro-apoptotic effects in an IGF2BP2-dependent manner and that targeting METTL3 alleviated the DN injury (99). A negative effect of METTL3 in DN was reported also by Tang et al. (100). METTL14 was also highly expressed in the kidneys of DN patients and HRGEC (high glucose-induced human renal glomerular endothelial cells). METTL14 worsened renal injury and inflammation was reported in db/db mice (101). Lu et al. also reported high levels of METTL14 in renal biopsy samples from patients with glomerulosclerosis and DN. Mice with podocyte-specific METTL14 deletion were then associated with improved glomerular function and alleviated podocyte injury compared to wild-type nephropathic mice (102). Also the third component of the MTC – WTAP – was reported to induce pyroptosis and inflammation in high glucose-treated HK-2 cells (103). Besides the methyltransferases, FTO was described to promote the progression of DN (104). However, several SNPs in the *FTO* gene were associated with a significantly lower risk of nephropathy in T2DM patients (62). Urine levels of m^6^A were decreased in patients with T2DM and even more with DN (105).

The existing data indicate that m^6^A machinery is affected in DN and that its dysregulation has a negative outcome on the progression of the pathology.

### 4.5 Liver

Liver disease ranks among notable causes of death in T2DM patients (106). Non-alcoholic fatty liver disease (NAFLD) is the most common chronic liver disease and is strongly associated with T2DM (107–109). The prevalence of this comorbidity among T2DM patients reaches up to 70% (110). It has been described that NAFLD is promoted by m^6^A modification dysregulation (111–116). Moreover, liver tissues from T2DM patients and mice on HFD showed elevated levels of m^6^A and also METTL3. Hepatocyte-specific knockout of *Mettl3* in mice then led to improved insulin sensitivity and decreased fatty acid synthesis (117). Jiang et al. also reported that baicalin – a flavonoid glycoside used in traditional Chinese medicine – suppressed T2DM-induced liver tumor progression in a METT3/m^6^A-dependent manner (118).

### 4.6 Eyes

Chronic exposure to hyperglycemia affects the microvasculature, eventually leading to diabetic retinopathy (DR), the main cause of blindness in the developed world. It has been described that m^6^A modification is regulated by various risk factors associated with DR, such as inflammation, oxidative stress, angiogenesis, or glucose and lipid metabolism (119). *FTO* polymorphism (rs8050136) was associated with a higher risk of DR (120). In retinal pigment epithelium (RPE) cells, high-glucose conditions down-regulated the expression of METTL3 on both transcript and protein levels. Further experiments showed that METTL3 overexpression alleviated the cytotoxic effects of high-glucose on RPE cells, while METTL3 depletion had the opposite effect (121). Conversely, diabetic stress-induced up-regulation of METTL3 and subsequent increase of m^6^A levels in human retinal pericytes and also mouse retinas. Specific depletion of METTL3 in pericytes suppressed diabetes-induced pericyte dysfunction and vascular complication *in vivo* (122). A recent study showed down-regulation of METTL3 in vitreous humor samples from patients with DR, a mouse model of DR, and also high glucose-induced human retinal microvascular endothelial cells (123).

Despite these conflicting data on METTL3 expression, it seems to be clear that epitranscriptomic regulations are affected in DR, but the exact role of m^6^A in the pathogenesis remains to be elucidated in the future.

### 4.7 Skin

Dysregulation of autophagy is a contributing factor for delayed wound healing in diabetic skin. YTHDC1, an m^6^A reader, has been described as a modulator of autophagy in diabetic keratinocytes which regulates the mRNA stability of an autophagy receptor (124). Interestingly, YTHDC1 interacted and cooperated with ELAVL1 (ELAV-like RNA binding protein 1), a well-established RNA stabilizer also linked to m^6^A methylation. It has been described previously that loss of m^6^A methylation enhances ELAVL1 RNA binding to increase RNA stability (125).

### 4.8 Blood

Decreased m^6^A methylation levels were detected in RNA isolated from the peripheral blood of T2DM patients and also diabetic rats (126, 127). In accordance with these results, significantly higher gene expression of *FTO* (and not *ALKBH5*) in peripheral blood from T2DM patients was detected (126). However, Onalan et al. (127) observed an up-regulated expression of both demethylases in venous blood samples from T2DM patients. The increased expression of FTO on both gene and protein levels was later confirmed by another study which pointed out the correlation between high FTO levels and T2DM severity (128). The gene expression of *FTO* was also up-regulated in white blood cells from T2DM patients compared to healthy individuals and the expression level of *FTO* was positively correlated with fasting glucose concentration (129). Besides erasers, *METTL3* mRNA was down-regulated in serum samples from T2DM patients (121). Progressively higher T2DM risk was associated with low serum IGF2BP3 levels (72).

Taken together, the content of m^6^A or its regulators in the peripheral blood may serve as novel potential biomarkers of T2DM in the future (126).

### 4.9 Treatment of T2DM: the role of m^6^A and m^6^Am modifications

Metformin is the first-line therapy for the treatment of T2DM, yet its molecular mechanisms of action are not fully understood (130, 131). The main effect of metformin treatment is inhibition of hepatic gluconeogenesis. At the molecular level, several mechanisms have been proposed to explain this phenomenon, such as inhibition of mitochondrial complex I activity, activation of AMPK, or increase in hepatocellular redox state due to inhibition of GPD2 (glycerol-3-phosphate dehydrogenase 2). The secondary effects of metformin treatment include an increase in muscle glucose uptake, a decrease in intestinal glucose absorption, and a change in the composition of the gut microbiome (130).

According to recent studies, metformin also affects epitranscriptomic regulations, including m^6^A machinery. Metformin was shown to reduce m^6^A methylation via the down-regulation of methyltransferase METTL3 in breast cancer cells (132). In hepatocellular carcinoma, metformin treatment was associated with METTL3 inhibition (133). Metformin also attenuated multiple myeloma cell proliferation and encouraged apoptosis by suppressing METTL3-mediated m^6^A methylation of its targets (134). Surprisingly, METTL3 expression was up-regulated after metformin treatment in adenocarcinoma cells (135). YTHDC2, a key m^6^A reader, is an important target of metformin in preventing the progression of vascular smooth muscle cell (VSMC) dysfunction under high glucose, a simulation of VSMC dysfunction caused by T2DM (136). Recently, Liao et al. (137) showed that metformin combats obesity by targeting FTO in an m^6^A-YTHDF2-dependent manner. This study suggests that metformin inhibited the protein expression of FTO, resulting in higher m^6^A methylation in mRNAs of crucial cell cycle regulators. The binding of YTHDF2 to modified transcripts then triggered mRNA decay and subsequent decrease of protein expression. In consequence, the mitotic clonal expansion process was blocked and adipogenesis was inhibited.

This fragmentary information suggests that metformin may both decrease and increase m^6^A methylation and that the target tissue or cell type may be the determining factor. However, *in vivo* studies focusing on the epitranscriptomic effect of metformin are needed to decipher this phenomenon, as the *in vivo* and *in vitro* response may also differ, especially if the primary target of metformin treatment is the liver. Despite these ambiguities, the association between epitranscriptomics and metformin is revealing itself, however, the role of m^6^A modification in the treatment of diabetes remains unclear.

## 5 Conclusion and perspectives

**Figure 4:**
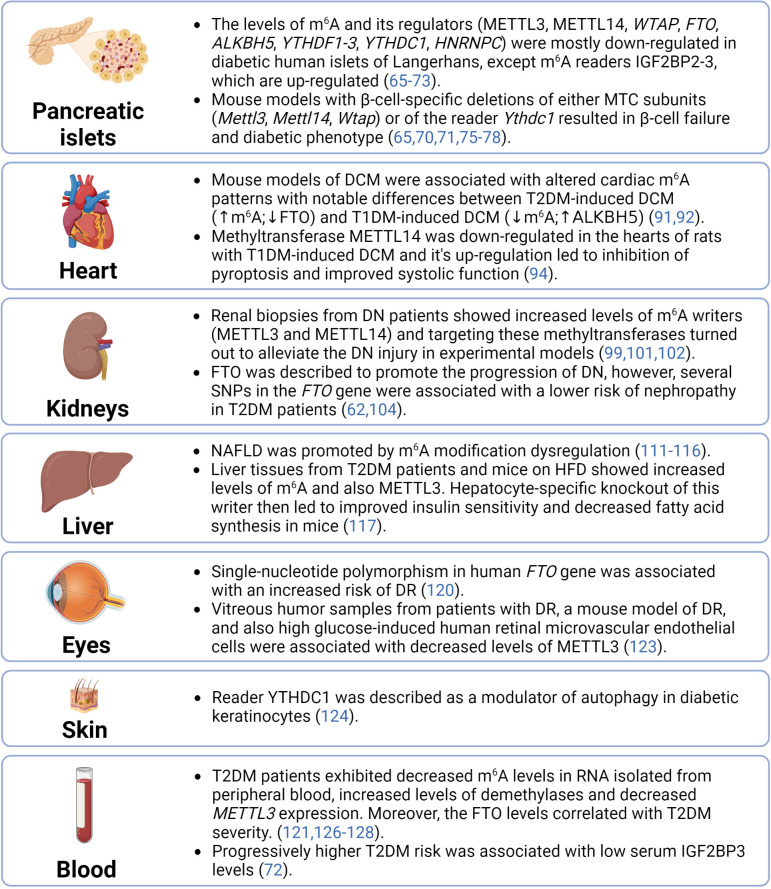
Summary of epitranscriptomic regulations in diabetic tissues. ALKBH5, AlkB family member 5; DCM, diabetic cardiomyopathy; DN, diabetic nephrophathy; DR, diabetic retinopathy; FTO, fat mass and obesity-associated; HFD, high-fat diet; HNRNPC, heterogeneous nuclear ribonucleoprotein C; IGF2BP2-3, insulin-like growth factor 2 mRNA binding proteins 2-3; m^6^A, N^6^-methyladenosine; METTL14, methyltransferase-like 14; METTL3, methyltransferase-like 3; MTC, multicomponent methyltransferase complex; NAFLD, non-alcoholic fatty liver disease; SNPs, single-nucleotide polymorphisms; T1DM, type 1 diabetes mellitus; T2DM, type 2 diabetes mellitus; WTAP, Willms’ tumor 1-associating protein; YTHDC1, YTH domain-containing protein 1; YTHDF1-3, YTH domain-containing family proteins 1-3.

The significant role of epitranscriptomics in cellular physiology and pathophysiology has been widely accepted by the scientific community in the past few years. However, despite the increased interest of researchers in RNA modifications, the complex epitranscriptomic regulations are still not fully understood. Our review focused on two of the most prevalent modifications – m^6^A and m^6^Am – in the pathogenesis of T2DM. The fragmental current knowledge indicates that diabetic tissues are associated with the dysregulation of epitranscriptomic machinery (summarized in **Figure 4**). However, it is essential to correctly distinguish whether these dysregulations contribute to the development of the disease or are merely a consequence of it. Several studies already showed that a deficiency of epitranscriptomic regulators can promote the pathological conditions typical for T2DM. Thus, targeting the epitranscriptomic regulations might have future applications in the clinic and consequently reduce the morbidity and mortality of T2DM patients.

## Author contributions

DB and SB drafted the article, LP-H and MH provided substantive revisions. All authors contributed to the article and approved the submitted version.
